# APOBEC3 selects V179I in HIV-1 reverse transcriptase to provide selective advantage for non-nucleoside reverse transcriptase inhibitor-resistant mutants

**DOI:** 10.3389/fviro.2022.919825

**Published:** 2022-07-14

**Authors:** Richa Dwivedi, Youya Wang, Christopher Kline, Douglas K. Fischer, Zandrea Ambrose

**Affiliations:** Department of Microbiology and Molecular Genetics, University of Pittsburgh School of Medicine, Pittsburgh, PA, United States

**Keywords:** HIV, reverse transcriptase (RT), drug resistance, non-nucleoside RT inhibitor (NNRTI), APOBEC3, humanized mouse model

## Abstract

The V179I substitution in human immunodeficiency virus type 1 (HIV-1) reverse transcriptase (RT) is selected in humans or mouse models treated with certain nonnucleoside reverse transcriptase inhibitors (NNRTIs). While it is often observed together with other NNRTI resistance mutations, V179I does not confer drug resistance. To understand how V179I arises during NNRTI treatment, we characterized it in HIV-1 molecular clones with or without the NNRTI resistance mutations Y181C or Y181V. While V179I alone did not confer resistance to any NNRTIs tested, when present with Y181C/V it enhanced drug resistance to some NNRTIs by 3- to 8-fold. In replication competition experiments in the presence of the NNRTI rilpivirine (RPV), V179I modestly enhanced Y181C HIV-1 or Y181V HIV-1 replication compared to viruses without V179I. As V179I arises from a G to A mutation, we evaluated whether it could arise due to host APOBEC3 deaminase activity and be maintained in the presence of a NNRTI to provide a selective advantage for the virus. V179I was detected in some humanized mice treated with RPV and was associated with G to A mutations characteristic of APOBEC3 activity. In RPV selection experiments, the frequency of V179I in HIV-1 was accelerated in CD4+ T cells expressing higher APOBEC3F and APOBEC3G levels. Our results provide evidence that V179I in HIV-1 RT can arise due to APOBEC-mediated G to A hypermutation and can confer a selective advantage to drug-resistant HIV-1 isolates in the presence of some NNRTIs.

## Introduction

The goals of antiretroviral drugs are to suppress replication of HIV-1 in infected individuals (antiretroviral therapy, ART) and to prevent infection of HIV-1 in uninfected individuals (preexposure prophylaxis, PrEP, and post-exposure prophylaxis, PEP). Nonnucleoside reverse transcriptase inhibitors (NNRTIs) comprise a class of antiretroviral drugs that target the HIV-1 enzyme reverse transcriptase (RT) that are used for both ART and PrEP. They inhibit reverse transcription by binding to a hydrophobic pocket near the polymerase active site, which prevents DNA polymerization (1). There are five NNRTIs currently approved by the U.S. Federal Drug Administration (FDA) for ART through daily oral dosing: nevirapine (NVP), efavirenz (EFV), etravirine (ETR), rilpivirine (RPV), and doravirine (DOR) (2). In 2021, the FDA approved monthly dosing of a long-acting injectable formulation of RPV (RPV LA) with the integrase inhibitor cabotegravir for HIV-1 maintenance therapy in individuals with virologic suppression based on results of the ATLAS and FLAIR phase III clinical trials (3). Recently, the World Health Organization recommended the use of vaginal rings containing the NNRTI dapivirine (DAP) as PrEP for women in light of positive results from the phase III ASPIRE and The Ring Study clinical trials (4, 5).

Due to high frequencies of circulating NNRTI-resistant isolates, NNRTIs are no longer recommended as first line ART in the U.S. (6). The more recently approved NNRTIs were developed to have more flexibility in binding to HIV-1 RT in an effort to inhibit variants that are resistant to NVP and EFV. The HIV-1 mutations conferring resistance to the diarylpyrimidines (ETR, RPV, or DAP) are similar (7, 8). Frequently selected resistance mutations of individuals failing therapy containing ETR or RPV include substitutions at E138, K101, and Y181 in RT (9–11). These mutations also were detected in women who seroconverted despite the use of DAP PrEP (12). Y181C is one of the most transmitted NNRTI resistance mutations globally, which confers broad NNRTI resistance (13), including low-level resistance (2- to 3-fold) to DAP, ETR, and RPV. Additional nucleotide changes to produce other NNRTI-associated mutations in combination with Y181C or to convert Y181C to Y181V lead to higher resistance to these drugs (7, 8).

V179 is a polymorphic residue in HIV-1 RT, particularly subtypes A and C, which is detected in treatment-naive individuals (14–20). Amino acid substitutions at 179, including V179I, increased in frequency in HIV-infected individuals treated with ART containing ETR or RPV (21–23). Despite its increased association with NNRTI treatment, the presence of baseline V179I prior to therapy did not necessarily correlate with virologic failure on ETR- or RPV-containing ART (10, 17, 24) and has little or no effect on NNRTI susceptibility (25, 26). V179I is often detected with other drug resistance mutations, such as Y181C (22, 23, 27–29).

The apolipoprotein B messenger RNA-editing enzyme catalytic polypeptide 3 (APOBEC3) family of mammalian proteins are cytidine deaminases that convert cytidine residues to uridines. APOBEC3G and APOBEC3F are packaged into HIV-1 particles and deaminate negative-sense viral DNA, resulting in G to A mutations in the viral positive-sense strand that can restrict infection (30). The antiviral activity of APOBEC3F/G is counteracted by the HIV-1 accessory protein Vif, which recruits APOBEC3F/G to the ubiquitin E3 ligase complex for proteasomal degradation (31). APOBEC3-mediated mutations have been shown to lead to HIV-1 mutations that confer resistance to RT inhibitors, including the RPV-resistant substitution E138K in RT (32–35).

In a recent humanized mouse study, we reported low frequencies of V179I in plasma HIV-1 RNA from mice infected with WT or Y181V HIV-1 after treatment with RPV-LA PrEP (36). In the present study, we sought to understand the selection advantage of V179I in HIV-1 by characterizing its effect on NNRTI resistance and replication of HIV-1 with RPV-resistant mutations. In addition, we addressed the possibility that V179I could arise by APOBEC3-mediated G to A mutation.

## Results

### V179I enhances Y181C/V HIV-1 resistance to ETR and DAP but not to other NNRTIs

**FIGURE 1:**
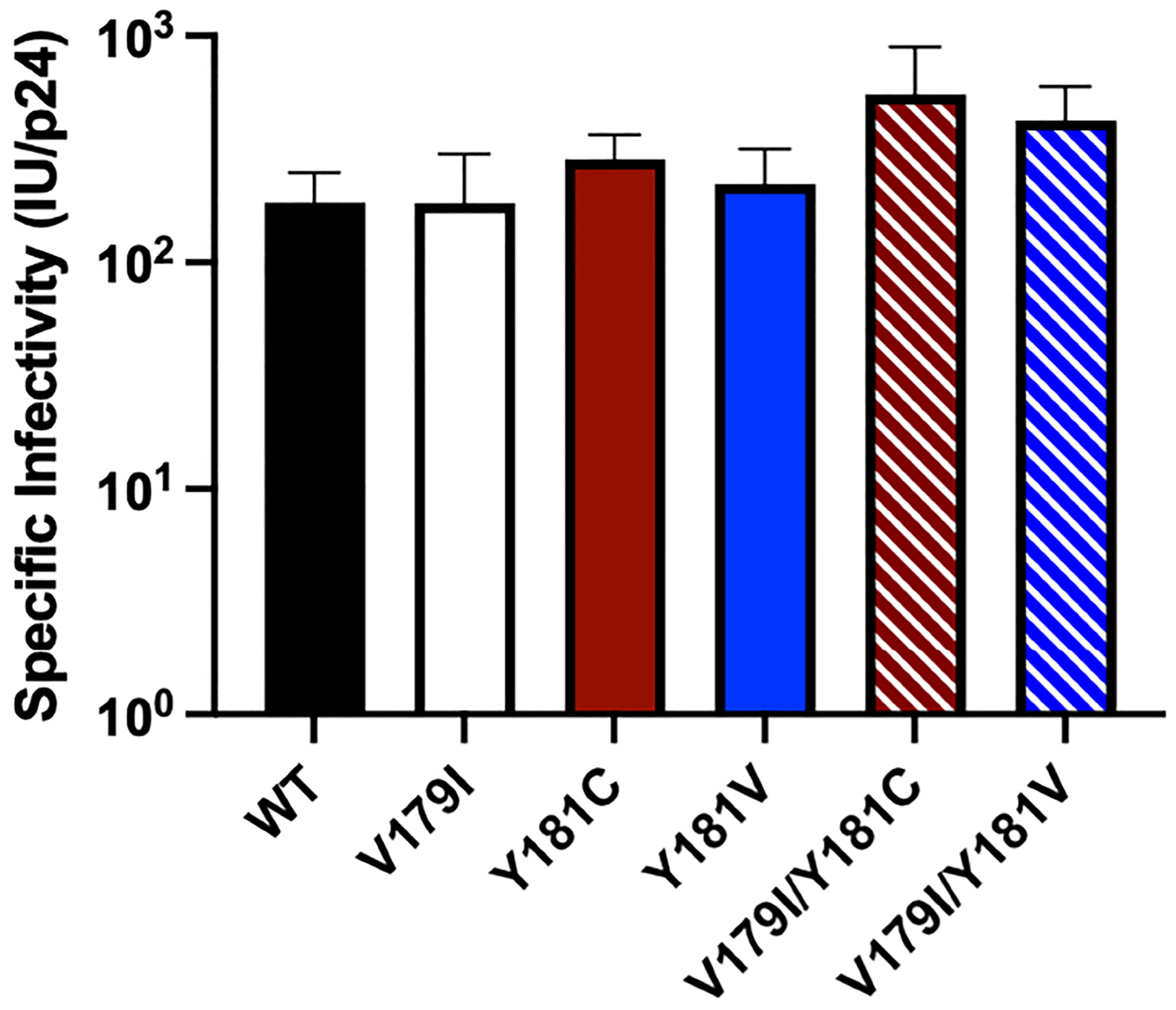
The specific infectivities of viruses used in drug susceptibility assays were determined by measuring titers (infectious units, IU) in GHOST cells and normalizing for p24 levels.

While V179I alone does not affect NNRTI susceptibility (25, 26), it has been shown to increase resistance in combination with other mutations (7, 9). Therefore, its effect in combination with Y181C/V was evaluated in the subtype B molecular clone used in our previous humanized mouse study (36). Drug susceptibility assays were performed with VSV-G pseudotyped wild-type (WT) HIV-1_NL4–3_ and molecular clones containing V179I, Y181C, and Y181V alone or in combination with multiple concentrations of EFV, DAP, ETR, RPV, and DOR. The specific infectivity of each virus was determined, which was similar for all viruses (Figure 1). The average 50% effective concentrations (EC_50_) and fold-changes compared to WT for the NNRTIs were calculated for the WT and mutant viruses (Table 1). Following previous convention, low-, intermediate- and high-level resistance were defined as a 2- to 8-fold, 8- to 20-fold, or >20-fold increase in EC_50_ for the mutants compared to WT HIV-1, respectively (8).

As expected, Y181C conferred low to intermediate resistance to all of the NNRTIs tested (2- to 9-fold; Table 1). In contrast, Y181V conferred high level resistance towards DAP, ETR, and RPV (735-, 64- and 35-fold, respectively) and low level resistance towards EFV and DOR (6- and 3-fold, respectively). An NRTI, FTC, was used as a control and, as expected, all of the viruses showed similar susceptibility (data not shown).

V179I alone did not confer drug resistance to any of the tested NNRTIs (Table 1), which is consistent with previous reports (25, 37). While V179I/Y181C HIV-1 susceptibility to EFV, RPV, and DOR did not differ from Y181C HIV-1, the addition of V179I enhanced Y181C HIV-1 resistance to DAP and ETR, with a change from 9- to 74-fold and 4- to 15-fold resistance, respectively. Similarly, V179I with Y181V only showed enhanced resistance to DAP (735- to 3579-fold) and ETR (64- to 167-fold) and no change for the other NNRTIs.

### V179I did not enhance E138K HIV-1 resistance to DAP or RPV

In the aforementioned humanized mouse study, we also observed the amino acid substitution E138K arising in HIV-1 RT in the same mice that also had detectable V179I (36). E138K is a mutation that is selected by and confers low-level resistance to DAP, ETR, and RPV (9, 38, 39). Although E138K was never detected on the same viral genomes with V179I, we investigated whether the combination of V179I and E138K in HIV-1 RT could enhance DAP or RPV resistance compared to E138K alone (Table 2). While E138K HIV-1 had 4-fold resistance to DAP and RPV, which was consistent with previous reports, the addition of V179I did not change virus susceptibility to either drug.

### V179I enhances replication of Y181C and Y181V HIV-1 in the presence of RPV

**FIGURE 2:**
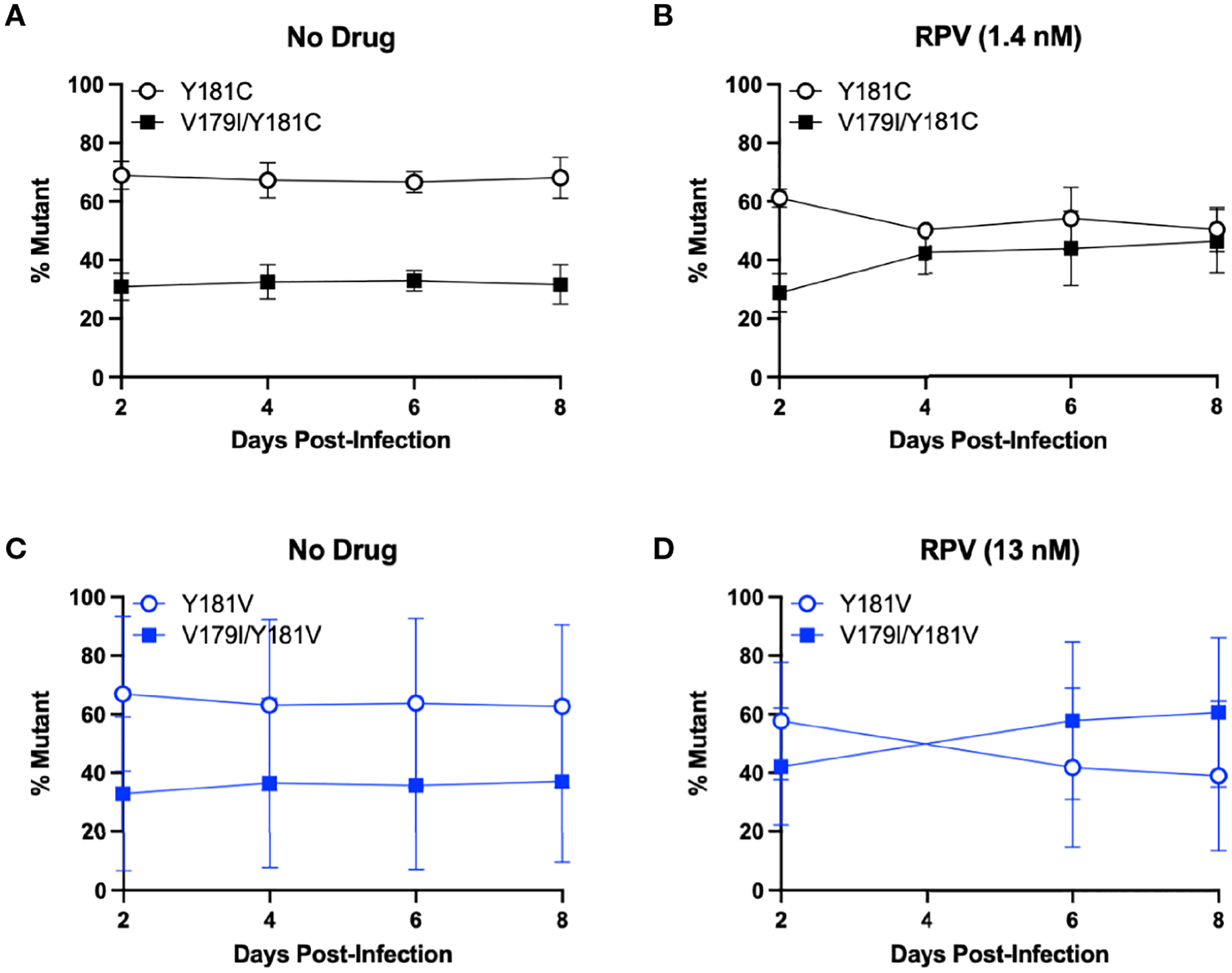
V179I enhances Y181C and Y181V HIV-1 replication in the presence of RPV. Competition assays were performed in HuT-R5 cells infected with Y181C/V HIV-1 with or without V179I at a 1:1 ratio. Cells were infected with Y181C HIV-1 and V179I/Y181C HIV-1 in the (**A**) absence or (**B**) presence of 1.4 nM RPV or Y181V HIV-1 and V179I/Y181V HIV-1 in the (**C**) absence or (**D**) presence of 13 nM RPV. The frequency of each virus in a culture was determined by PrimerID MiSeq over 8 days. Data represent averages from 2 independent experiments with error bars representing standard errors of the mean.

Surprisingly, V179I in HIV-1 RT is associated with virologic failure to RPV-containing ART, especially together with Y181C (21, 22), but it does not confer or enhance RPV resistance. We hypothesized that V179I could improve Y181C HIV-1 replication in the presence of RPV. As we also detected V179I in HIV-1 RT in humanized mice treated with RPV LA and challenged with Y181V HIV-1 (36), we also hypothesized that V179I/Y181V HIV-1 could have improved replication in the presence of RPV. To determine whether the double mutants could replicate better than Y181C/V HIV-1, growth competition assays were performed in the absence or presence of RPV, in which each culture contained a pair of viruses at 50% frequency based on virus titers. Replication was compared for Y181C HIV-1 and V179I/Y181C HIV-1 in the absence of drug by PrimerID MiSeq (Figure 2A). Y181C HIV-1 was detected at a frequency of approximately 65% in an average of two independent experiments, while V179I/Y181C HIV-1 was present at 35% frequency, likely due to an infection ratio slightly different than 1:1. Over the course of infection, no change in frequencies of the two viruses occurred, suggesting that V179I did not improve Y181C HIV-1 replication in the absence of drug. The frequency of V179I/Y181C HIV-1 increased to approximately 50% frequency in the presence of RPV (Figure 2B). These results suggest that the double mutant had slightly improved replication in the presence of RPV.

The same competition experiment was performed for Y181V HIV-1 and V179I/Y181V HIV-1. In the absence of drug, Y181V HIV-1 was present at approximately 65% frequency and the double mutant was at 35% frequency in an average of two independent experiments (Figure 2C). Similar to the Y181C experiment, this ratio was maintained over 8 days, suggesting that V179I did not improve Y181V HIV-1 replication in the absence of drug. However, in the presence of RPV, V179I/Y181V HIV-1 increased over time, particularly in one replicate (Figure 2D), suggesting that V179I together with Y181V gave HIV-1 a slight selective advantage over Y181V HIV-1 alone.

### Selection of V179I in HIV-1 is associated with G to A mutations *in vivo*

**FIGURE 3:**
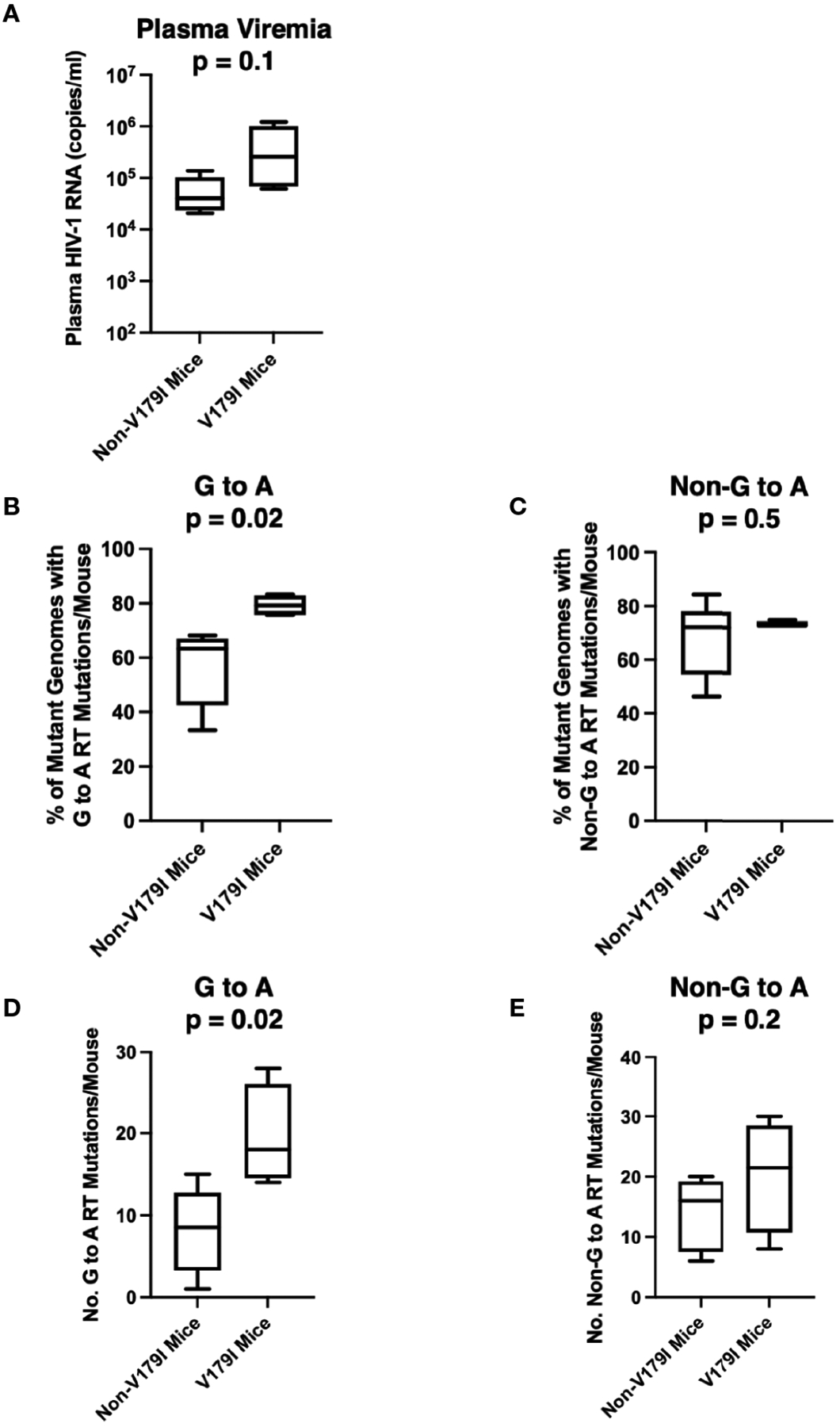
Selection of V179I in HIV-1 is associated with G to A mutations in infected humanized mice treated with RPV LA. HIV-1 RT single-genome sequences (42–47 per mouse) were analyzed in 10 humanized mice made from Donor 255 that were infected with WT or Y181V HIV-1 after treatment with RPV LA. (**A**) The HIV-1 RNA levels in the plasma at the time of necropsy are shown for the group of animals with (n=4) or without (n=6) detectable V179I in their viruses. The percentages of (**B**) G to A mutations or (**C**) non-G to A mutations were determined in each group of mice. The total number of (**D**) G to A or (**E**) non-G to A RT mutations were determined in mice with or without detectable V179I in their viruses. Box and whisker plot error bars indicate minimum and maximum values. Two-tailed unpaired t tests were performed between both groups for each analysis.

V179I is caused by a G to A point mutation. We hypothesized that V179I could arise due to mutations induced by APOBEC3F/G. V179I was detected inHIV-1 single-genome sequences from4 of 16 humanized mice treated with RPV LA PrEP that became infected after HIV-1 challenge (36).All four animals were made from tissues taken from one particular donor (Donor 255; 4/10 animals). To determine whether detection of V179I in HIV-1 RT from RPV LA-treated humanized mice was associated with more G to A mutations, we analyzed single-genome sequences (42–47 per animal) encoding the first 277 residues of RT isolated from plasma HIV-1 obtained from Donor 255 animals at 5–10 weeks after infection. G to A and non-G to A mutations were identified and compared to the sequences of the molecular clones used to challenge the mice (WT HIV-1 or Y181V HIV-1). Despite the animals having similar plasma viremia levels, suggesting similar *in vivo* replication of the viruses (Figure 3A), the group of mice with no detectable V179I HIV-1 had a significantly lower frequency of HIV-1 genomes that contained G to A mutations in the RT coding region than the group of mice with V179I HIV-1 (Figure 3B). In contrast, the frequency of other mutations (not G to A) in RT was similar in both groups of animals (Figure 3C). In addition, the number of G to A mutations in all of the single-genome sequences per mouse were significantly higher in those with V179I HIV-1 compared to those with out V179IHIV-1(Figure 3D).However, the total number of other mutations was similar in both groups (Figure 3E). In the mice with detectable V179I, two of 180 single-genome sequences had stop codons, but neither of these also had the mutation conferring the V179I substitution. These results suggest that in mice infected despite RPV LA treatment, V179I in HIV-1 was associated with greater G to A mutations in RT. APOBEC3F/G enzyme packaging in virions is stochastic and this likely results in variation in the frequency of G to A mutations.

### APOBEC3F/G selects V179I in HIV-1 RT in the presence of RPV *in vitro*

**FIGURE 4:**
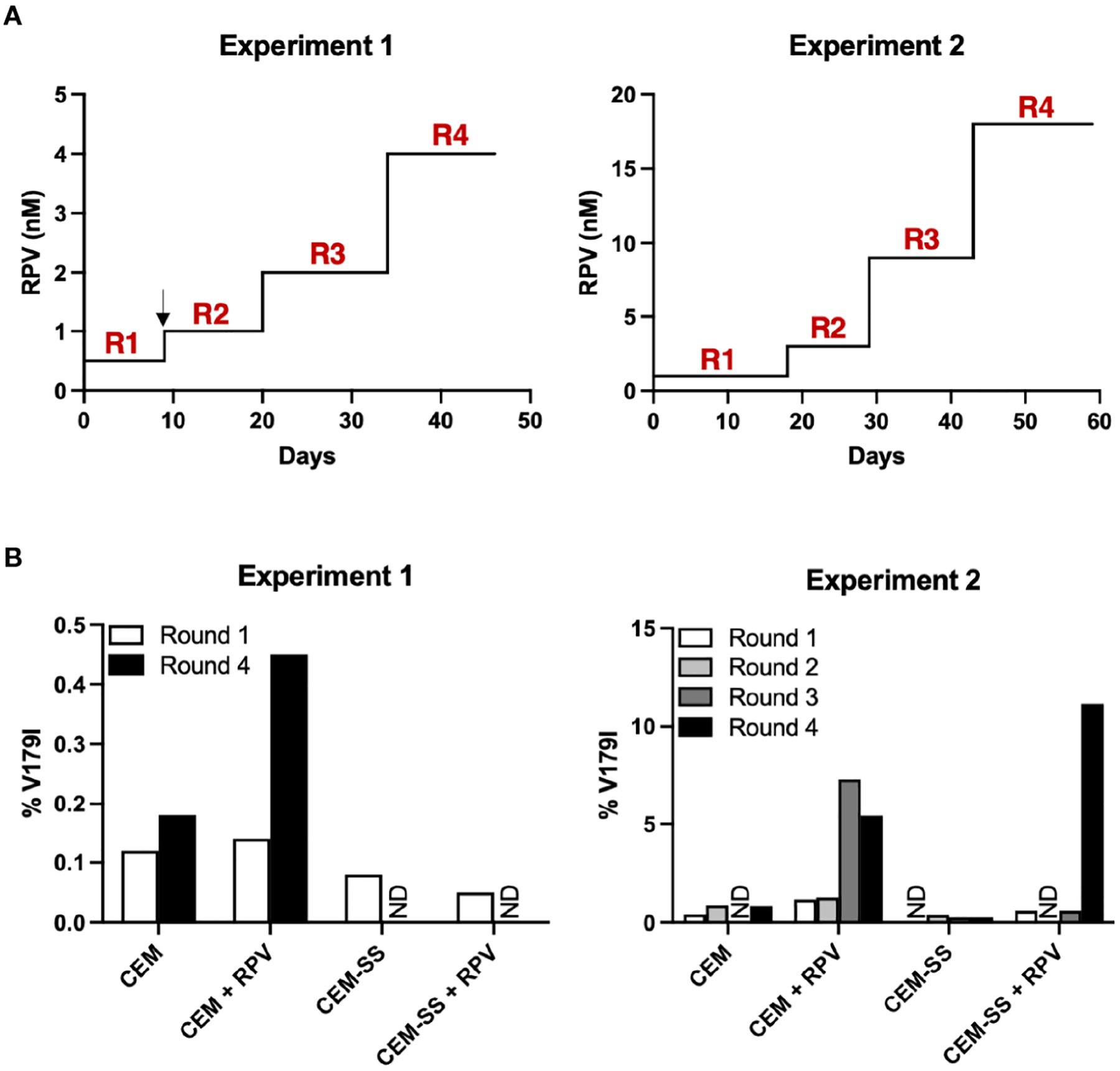
APOBEC3F/G selects V179I in HIV-1 RT in the presence of RPV in cells. (**A**) In two independent experiments, Y181C HIV-1 was grown in CEM cells or CEM-SS cells for four rounds with or without increasing concentrations of RPV (0.5 – 4 nM in Experiment 1; 1 – 18 nM in Experiment 2). The black arrow denotes when selection culture supernatants were frozen and restarted. (**B**) Single-genome sequencing by PrimerID MiSeq (1,514 and 628 average genomes for Experiments 1 and 2, respectively) of the region encoding the NNRTI-binding pocket of RT was performed on viral RNA isolated at the end of each round of selection. The frequencies of V179I in HIV-1 are shown for each culture. ND indicates that genomes encoding V179I were not detected.

CEM-SS cells are derived from the CEM CD4+ T cell line, but they express 28- and 12-fold lower APOBEC3F and APOBEC3G, respectively (40). To determine whether APOBEC3F/G-mediated editing could select V179I in HIV-1 RT in the presence of RPV, CEM cells and CEM-SS cells were infected with Y181C HIV-1 and cultured in the presence or absence of 0.5 – 1 nM RPV in two independent experiments. When HIV-1 replicated to relatively high levels, as indicated by syncytia formation and cell death, the concentration of RPV was increased 2- to 3-fold (Figure 4A). At least 98.8% of the HIV-1 population in the cultures still encoded Y181C during the course of selection (46 – 59 days). After the first round of selection in the first experiment, 0.12 – 0.14% of HIV-1 genomes from CEM cells, with or without RPV selection, encoded V179I (Figure 4B, left). In the presence of RPV, V179I rose to nearly 0.5% frequency in CEM cells. In contrast, 0.05 – 0.08% of HIV-1 genomes isolated in CEM-SS cells after round 1 encoded V179I. V179I was not detected after four rounds of selection in CEM-SS cells. In the second experiment, the frequency of V179I was below 0.9% in the cultures after rounds 1 and 2 with the exception of virus grown in CEM cells in the presence of RPV, in which the mutation was detected at 1.2 – 1.3% (Figure 4B, right). After rounds 3 and 4, the frequency of V179I rose to 7.3% and 5.4% in CEM cells + RPV but remained < 0.8% in the other cultures. Surprisingly, genomes containing V179I rose to 11.2% frequency in CEM-SS cells after the RPV concentration was increased to 18 nM. These results suggest that APOBEC3F/G accelerates selection of V179I in HIV-1 RT in the presence of RPV and that V179I provides a selective advantage for the virus.

## Discussion

We detected V179I in HIV-1 RT from humanized mice that became infected during RPV LA PrEP (36). Similarly, V179I HIV-1 is selected in people treated with ART containing ETR or RPV (21–23). Previous *in vitro* experiments showed that ETR and DAP selected for V179 substitutions, including V179I, in Y181C HIV-1 (22, 27, 39). Likewise, V179I was selected in mice infected with Y181V HIV-1 after RPV LA PrEP, suggesting that V179I provides a selective advantage for Y181C HIV-1 or Y181V HIV-1 in the presence of NNRTIs.

Similar to previous studies (25, 26), we observed that V179I alone did not confer drug resistance to any FDA-approved NNRTIs. However, V179I enhanced ETR and DAP resistance for Y181C HIV-1 4- to 8-fold, respectively, and Y181V HIV-1 3-to 5-fold, respectively. In contrast, V179I did not alter NNRTI susceptibility when present together with E138K in HIV-1 RT, which was also selected in HIV-1 isolated from our RPV-treated mice but not on the same genomes as V179I. V179I also did not affect Y181C/V HIV-1 sensitivity to non-diarylpyrimidine NNRTIs EFV and DOR. These results suggest a selective advantage for V179I in Y181C/V HIV-1 during replication in the presence of ETR or DAP.

Surprisingly, V179I did not enhance Y181C/V HIV-1 resistance to RPV, which suggested that this mutation provides a different selective advantage in the presence of RPV. While Y181C/V HIV-1 did not replicate differently than V179I/Y181C HIV-1 or V179I/Y181V HIV-1 in the absence of inhibitor, the double mutant viruses replicated slightly more efficiently in relatively low concentrations (2-fold above the EC_50_) of RPV. Additionally, V179I was selected in infectedCEM and CEM-SS cells in the presence of high levels of RPV, suggesting that it provides a selective advantage. Y181C HIV-1 can be rapidly selected by NNRTIs and has relatively high prevalence (13). In contrast, Y181V HIV-1 confers higher resistance to RPV (and ETR and DAP) and has similar replication to WT HIV-1 (36), but it is less prevalent in HIV-infected individuals, likely due to the requirement of two base changes (41). Nevertheless, V179I selection in both Y181C HIV-1 and Y181V HIV-1 provides a replication advantage for the double mutant in the presence of RPV.

The diarylpyrimidine NNRTIs have similar structures and binding modes in the HIV-1 RT NNRTI-binding pocket as well as have similar resistance profiles (42, 43). Upon binding, the pyrimidine ring of DAP, ETR, and RPV is positioned between L100 and V179 of HIV-1 RT, allowing interactions with K101 of the p66 subunit and E138 of the p51 subunit (42–44). However, distinct differences are seen in the crystal structures of ETR and RPV binding to WT RT (44). For example, the pyrimidine ring of RPV is located closer to E138 than that of ETR. In addition, the methylphenylacrylonitrile group of RPV interacts more strongly than the dimethylcyanophenyl group of ETR to residues Y181 and Y188 of RT. It is possible that one or both of these variations in binding can account for the phenotypic differences that we observe in V179I effects on DAP/ETR and RPV susceptibilities.

V179I is a polymorphic residue that is observed in treatment-naive HIV-infected individuals (14–20) but that has higher prevalence after NNRTI exposure, particularly the diarylpyrimidines. APOBEC3 has been shown to lead to HIV-1 mutations, which can lead to a replication advantage for the virus, such as conferring resistance to RT inhibitors like E138K (32–35). We noted that V179I is caused by a G to A mutation and hypothesized that it could arise in the presence or absence of NNRTI treatment due to APOBEC3F/G. Indeed, V179I was associated with higher frequencies of G to A mutations in HIV-1 isolated from RPV-treated mice. In addition, we directly investigated the ability of APOBEC3F/G to increase V179I selection in the presence of RPV using CEM and CEM-SS cells, which differ in their expression levels of these host factors. V179I was selected more rapidly in the presence of RPV in CEM cells that express higher APOBEC3F/G levels compared to CEM-SS cells that have low APOBEC3F/G expression. Thus, V179I could arise in HIV-1 RT due to APOBEC3 even prior to therapy, allowing it to persist or increase in frequency in individuals treated with DAP, ETR, or RPV due to improved replication and/or drug resistance. The importance of APOBEC3-mediated emergence of V179I over stochastic mutations caused by RT may vary in different individuals and cannot be definitively assessed in this study.

The results from this study provide insight in how and why the enigmatic V179I substitution is selected in HIV-1 RT during NNRTI treatment. While this residue change does not directly affect HIV-1 susceptibility to NNRTIs, and also likely NNRTI binding to RT, it appears to provide better replication in the presence of diarylpyrimidine NNRTIs by either enhancement of resistance or replication. This information could be useful in designing better RT inhibitors that can still inhibit prevalent NNRTI-resistant HIV-1 variants.

## Materials and methods

### Cells and antiretroviral inhibitors

293T and TZM-bl cells were cultured in Dulbecco’s modified Eagle’s medium (Thermo Fisher Scientific) supplemented with 10% fetal bovine serum (FBS; Atlanta Biologicals), 100 U/ml penicillin, 100 mg/ml streptomycin, and 0.292 mg/ml of L-glutamine (P/S/G; Thermo Fisher Scientific). GHOST-R3/X4/R5 cells (45) were maintained in the medium described above with the addition of 100 mg/ml of G418 (Thermo Fisher Scientific), 100 mg/ml hygromycin (Thermo Fisher Scientific), and 0.5 mg/ml puromycin (EMD Millipore). HuT-R5 cells (46) were cultured in RPMI 1640 medium (Invitrogen) supplemented with 10% FBS, P/S/G, 500 mg/ml G418, and 0.5 mg/ml puromycin. CEM and CEM-SS cells (47) were cultured in RPMI 1640 medium with 10% FBS and P/S/G. All cells were grown at 37°C in 5% CO_2_.

EFV, ETR, FTC, and RPV were obtained from the AIDS Reagent Program, Division of AIDS, National Institutes of Health. DAP and DOR were obtained from Dr. Nicolas Sluis-Cremer. Drug stocks were made in dimethyl sulfoxide (DMSO) and diluted to final concentrations in cell culture medium.

### Generation of WT and mutant HIV-1

The RT coding region of the *pol* gene of plasmids encoding the HIV-1_NL4–3_ with a deletion in *env* and the luciferase gene in place of *nef* (NLdE-luc) (48) or full-length HIV-1_NL4–3_ or HIV-1_NL4-BAL_ proviruses were amplified by PCR and were subcloned into pCR2.1-TOPO (Life Technologies). RT mutations E138K, V179I, Y181C, Y181V, E138K/V179I, V179I/Y181C, and V179I/Y181V were introduced into these plasmids using the QuikChange II XL site-directed mutagenesis kit (Agilent Technologies). After verification by Sanger sequencing, the mutated fragment was cloned into the plasmids.

WT and mutant viruses were produced by transfection of 293T cells using Lipofectamine 2000 (Thermo Fisher Scientific). NLdE-luc viruses were pseudotyped with VSV-G, using the pLVSV-G plasmid. Virus-containing supernatants were harvested 48h after transfection.

### Measurement of HIV-1 infectivity

Viruses were titered on GHOST R3/X4/R5 cells using an Accuri C6 flow cytometer (BD Biosciences), as previously described (45). NLdE-luc viruses were also assessed for p24 content *via* ELISA (XpressBio).

### Drug susceptibility assays

Susceptibility assays for WT and mutant HIV-1 (NLdE-luc) with NNRTIs DAP, DOR, EFV, ETR, and RPV as well as the NRTI FTC were performed in TZM-bl cells as previously described (49). Briefly, TZM-bl cells (2 × 10^4^ cells/well of a 96-well plate) were incubated with or without dilutions of drugs and infected with each virus at a multiplicity of infection (MOI) of0.02 in phenol red-free medium. After 48h, luciferase was measured using the Britelite Plus Reporter Gene Assay System (PerkinElmer). Infectivity was measured by luciferase expression in the cells, with uninfected cells set as background and infected cells in the absence of inhibitor set as 100%. Experiments were performed in triplicate in 3 or 4 independent experiments. The 50% effective concentration (EC_50_) was calculated in Prism (GraphPad) using nonlinear regression for each experiment.

### HIV-1 competition assays

Competition experiments were performed comparing Y181C and V179I/Y181C HIV-1_NL4–3_ or Y181V and V179I/Y181V HIV-1_NL4–3_ in two independent experiments, as previously described (50). Briefly, HuT-R5 cells were co-infected with each virus at a 1:1 ratio with a total MOI of 0.004 for 2h at 37°C. The cells were washed in phosphate buffered saline and resuspended in media with or without RPV. The concentration of RPV used was approximately 2-fold above the EC_50_ for Y181C HIV-1 or Y181V HIV-1 (with or without V179I): 1.4 nM and 13 nM, respectively. Cultures were split every 2 days for 8 days. HIV-1 RNA was isolated from cell supernatants at each time point using the RNeasy kit (Qiagen). PrimerID MiSeq was performed to sequence part of the RT coding region as described previously (50). The average number of paired end reads for each flask in Experiment 1 was 8,927 copies (range from 2,708 to 14,532 copies) and in Experiment 2 was 483 copies (range from 80 to 1054). The relative abundance of each mutant was calculated by dividing the number of mutant sequences by the total number of consensus sequences at each time point. Unexpected variants at 179 (i.e., not V or I) were present at low levels (0–1% of the total sequences in each sample) and were not maintained at multiple time points.

### RPV selection assays

Selections of Y181C HIV-1_NL4–3_ were performed in CEM cells and CEM-SS cells. CEM and CEM-SS cells were infected with Y181C HIV-1 at a MOI of 0.1 in the absence or presence of 0.5 nM RPV (Experiment 1) or 1 nM RPV (Experiment 2). Cultures were split every 2–3 days. When significant cytopathic effects were observed, cells were pelleted and supernatant was collected from each flask. Each culture was replenished with new cells and fresh medium with or without 2- to 3-fold higher RPV concentrations. The selection process was repeated for 4 rounds. Due to the COVID-19 pandemic, supernatants from cultures after round 1 in Experiment 1 were frozen at −80 C and were used to infect new cells in round 2. HIV-1 RNA was isolated from supernatant collected at the last time point from each round of selection using the RNeasy kit. PrimerID MiSeq for the RT coding region was performed as previously described (50) to determine the frequencies of amino acid substitutions at residues 179 and 181. The average number of paired end reads for each flask was 1,514 copies (range from 466 to 4,307 copies) for Experiment 1 and 628 copies (range from 173 to 1,222 copies) for Experiment 2. The relative abundance of each mutant was calculated by dividing the number of mutant sequences by the total number of consensus sequences at each time point. Unexpected variants were present at low levels (≤ 1% of the total sequences in each sample).

### RPV LA administration to humanized mice

We previously evaluated the ability of RPV LA to prevent vaginal transmission of WT, Y181C, or Y181V HIV-1 in humanized mice (36). Female immunodeficient NSG (NOD.*Cg-Prkdc*^*scid*^*Il2rgtm1*^*Wjl*^/SzJ) mice from Jackson Laboratory were implanted with fetal thymus, liver, and CD34 + cells obtain from the University of Pittsburgh Biospecimen Core with an Institutional Review Board-approved Honest Broker System. Mice (n = 30) were injected intramuscularly with 150 mg/kg RPV LA and challenged 1 or 7 days later with WT or mutant HIV-1_NL4-BAL_ and compared to untreated and challenged mice (n = 26). Of the RPV LA-treated mice, sixteen became infected as determined by detection of HIV-1 RNA in the plasma, as described previously (51). HIV-1 single-genome sequencing was performed on plasma HIV-1 RNA at necropsy, as previously described (52) to identify mutations in HIV-1 *pol*. A total of 715 sequences were obtained from these mice, of which 6 had stop premature stop codons. Two-tailed unpaired t tests were performed in Prism to compare viremia and mutations between groups.

## Figures and Tables

**TABLE 1:** WT and mutant HIV-1 susceptibility to NNRTIs.

	EFV EC_50_ (nM)	EFV Fold-change	DAP EC_50_ (nM)	DAP Fold-change	ETR EC_50_ (nM)	ETR Fold-change	RPV EC_50_ (nM)	RPV Fold-change	DOR EC_50_ (nM)	DOR Fold-change
**WT**	0.65 ± 0.30	-	0.26 ± 0.13	-	0.83 ± 0.23	-	0.19 ± 0.06	-	1.4 ± 0.28	-
**V179I**	0.79 ± 0.04	1	0.61 ± 0.21	2	0.87 ± 0.09	1	0.24 ± 0.09	1	0.88 ± 0.04	1
**Y181C**	2.3 ± 0.50	4	3.1 ± 1.6	9	2.3 ± 0.35	4	0.58 ± 0.31	3	2.4 ± 0.07	2
**V179I/Y181C**	2.3 ± 0.42	4	17 ± 1.4	74	14 ± 4.8	15	0.77 ± 0.11	4	2.3 ± 0.07	2
**Y181V**	3.4 ± 0.78	6	105 ± 17	735	54 ± 6.3	64	6.6 ± 2.1	35	4.6 ± 0.14	3
**V179I/Y181V**	3.0 ± 0.57	5	405 ± 261	3579	154± 18	167	6.7 ± 1.1	33	4.6 ± 0.28	3

**TABLE 2:** WT and E138K/V179I HIV-1 susceptibility to NNRTIs.

	DAP EC_50_ (nM)	DAP Fold-change	RPV EC_50_ (nM)	RPV Fold-change
**WT**	0.70 ± 0.00		0.21 ± 0.01	
**E138K**	2.6 ± 0.26	4	0.71 ± 0.02	4
**E138K/V179I**	3.2 ± 0.67	5	0.50 ± 0.05	2

## Data Availability

The raw data supporting the conclusions of this article will be made available by the authors, without undue reservation.

## References

[1] Das K, Martinez SE, Bauman JD, Arnold E. Hiv-1 reverse transcriptase complex with dna and nevirapine reveals non-nucleoside inhibition mechanism. Nat Struct Mol Biol (2012) 19(2):253–9. doi: 10.1038/nsmb.2223 22266819 PMC3359132

[2] Wang Y, De Clercq E, Li G. Current and emerging non-nucleoside reverse transcriptase inhibitors (Nnrtis) for hiv-1 treatment. Expert Opin Drug Metab Toxicol (2019) 15(10):813–29. doi: 10.1080/17425255.2019.1673367 31556749

[3] Rizzardini G, Overton ET, Orkin C, Swindells S, Arasteh K, Gorgolas Hernandez-Mora M, Long-acting injectable cabotegravir + rilpivirine for hiv maintenance therapy: week 48 pooled analysis of phase 3 atlas and flair trials. J Acquir Immune Defic Syndr (2020) 85(4):498–506. doi: 10.1097/QAI.0000000000002466 33136751 PMC7592884

[4] Baeten JM, Palanee-Phillips T, Brown ER, Schwartz K, Soto-Torres LE, Govender V, Use of a vaginal ring containing dapivirine for hiv-1 prevention in women. New Engl J Med (2016) 375(22):2121–32. doi: 10.1056/NEJMoa1506110 26900902 PMC4993693

[5] Nel A, van Niekerk N, Kapiga S, Bekker LG, Gama C, Gill K, Safety and efficacy of a dapivirine vaginal ring for hiv prevention in women. New Engl J Med (2016) 375(22):2133–43. doi: 10.1056/NEJMoa1602046 27959766

[6] U.S. Departmentof Health and Human Services. Panel on antiretroviral guidelines for adults and adolescents. In: Guidelines for the use of antiretroviral agents in adults and adolescents with hiv. Washington, D.C (2020). Available at: https://clinicalinfo.hiv.gov/sites/default/files/inline-files/AdultandAdolescentGL.pdf.

[7] Azijn H, Tirry I, Vingerhoets J, de Béthune M-P, Kraus G, Boven K, Tmc278, a next-generation nonnucleoside reverse transcriptase inhibitor (Nnrti), active against wild-type and nnrti-resistant hiv-1. Antimicrobial Agents chemotherapy (2010) 54(2):718–27. doi: 10.1128/AAC.00986-09 19933797 PMC2812151

[8] Giacobbi NS, Sluis-Cremer N. *In vitro* cross-resistance profiles of rilpivirine, dapivirine, and miv-150, nonnucleoside reverse transcriptase inhibitor microbicides in clinical development for the prevention of hiv-1 infection. Antimicrobial Agents Chemother (2017) 61(7):e00277–17. doi: 10.1128/AAC.00277-17 PMC5487620 28507107

[9] Rimsky L, Vingerhoets J, Van Eygen V, Eron J, Clotet B, Hoogstoel A, Genotypic and phenotypic characterization of hiv-1 isolates obtained from patients on rilpivirine therapy experiencing virologic failure in the phase 3 echo and thrive studies: 48-week analysis. J Acquir Immune Defic Syndr (2012) 59(1):39–46. doi: 10.1097/QAI.0b013e31823df4da 22067667

[10] Tambuyzer L, Vingerhoets J, Azijn H, Daems B, Nijs S, de Bethune MP, Characterization of genotypic and phenotypic changes in hiv-1-infected patients with virologic failure on an etravirine-containing regimen in the duet-1 and duet-2 clinical studies. AIDS Res Hum Retroviruses (2010) 26(11):1197–205. doi: 10.1089/aid.2009.0302 20854144

[11] Vingerhoets J, Tambuyzer L, Azijn H, Hoogstoel A, Nijs S, Peeters M, Resistance profile of etravirine: combined analysis of baseline genotypic and phenotypic data from the randomized, controlled phase iii clinical studies. AIDS (2010) 24(4):503–14. doi:10.1097/QAD.0b013e32833677ac 20051805

[12] Riddler SA, Balkus JE, Parikh UM, Mellors JW, Akello C, Dadabhai S, Clinical and virologic outcomes following initiation of antiretroviral therapy among seroconverters in the mtn-020/aspire phase iii trial of the dapivirine vaginal ring. Clin Infect Dis (2019) 69(3):523–9. doi: 10.1093/cid/ciy909 30346511 PMC6637274

[13] Rhee SY, Blanco JL, Jordan MR, Taylor J, Lemey P, Varghese V, Geographic and temporal trends in the molecular epidemiology and genetic mechanisms of transmitted hiv-1 drug resistance: an individual-patient- and sequence-level meta-analysis. PLoS Med (2015) 12(4):e1001810. doi: 10.1371/journal.pmed.1001810 25849352 PMC4388826

[14] Ceccherini-Silberstein F, Svicher V, Sing T, Artese A, Santoro MM, Forbici F, Characterization and structural analysis of novel mutations in human immunodeficiency virus type 1 reverse transcriptase involved in the regulation of resistance to nonnucleoside inhibitors. J Virol (2007) 81(20):11507–19. doi: 10.1128/JVI.00303-07 17686836 PMC2045529

[15] Grossman Z, Istomin V, Averbuch D, Lorber M, Risenberg K, Levi I, Genetic variation at nnrti resistance-associated positions in patients infected with hiv-1 subtype c. AIDS (2004) 18(6):909–15. doi: 10.1097/00002030-200404090-00008 15060438

[16] Hsu LY, Subramaniam R, Bacheler L, Paton NI. Characterization of mutations in Crf01_Ae virus isolates from antiretroviral treatment-naive and -experienced patients in singapore. J Acquir Immune Defic Syndr (2005) 38(1):5–13. doi: 10.1097/00126334-200501010-00002 15608517

[17] Lambert-Niclot S, Charpentier C, Storto A, Fofana DB, Soulie C, Fourati S, Prevalence of pre-existing resistance-associated mutations to rilpivirine, emtricitabine and tenofovir in antiretroviral-naive patients infected with b and non-b subtype hiv-1 viruses. J Antimicrobial chemotherapy (2013) 68(6):1237–42. doi: 10.1093/jac/dkt003 23361642

[18] Pillay C, Bredell H, McIntyre J, Gray G, Morris L. Hiv-1 subtype c reverse transcriptase sequences from drug-naive pregnant women in south africa. AIDS Res Hum Retroviruses (2002) 18(8):605–10. doi: 10.1089/088922202753747950 12036490

[19] Turner D, Brenner B, Moisi D, Detorio M, Cesaire R, Kurimura T, Nucleotide and amino acid polymorphisms at drug resistance sites in non-b-subtype variants of human immunodeficiency virus type 1. Antimicrob Agents Chemother (2004) 48(8):2993–8. doi: 10.1128/AAC.48.8.2993-2998.2004 15273111 PMC478480

[20] Vergne L, Stuyver L, Van Houtte M, Butel C, Delaporte E, Peeters M. Natural polymorphism in protease and reverse transcriptase genes and in vitro antiretroviral drug susceptibilities of non-b hiv-1 strains from treatment-naive patients. J Clin Virol (2006) 36(1):43–9. doi: 10.1016/j.jcv.2006.01.012 16563858

[21] Anta L, Llibre JM, Poveda E, Blanco JL, Alvarez M, Perez-Elias MJ, Rilpivirine resistance mutations in hiv patients failing non-nucleoside reverse transcriptase inhibitor-based therapies. AIDS (2013) 27(1):81–5. doi: 10.1097/QAD.0b013e3283584500 22842995

[22] Asahchop EL, Wainberg MA, Oliveira M, Xu H, Brenner BG, Moisi D, Distinct resistance patterns to etravirine and rilpivirine in viruses containing nonnucleoside reverse transcriptase inhibitor mutations at baseline. AIDS (2013) 27(6):879–87. doi: 10.1097/QAD.0b013e32835d9f6d 23262501

[23] Marcelin AG, Descamps D, Tamalet C, Cottalorda J, Izopet J, Delaugerre C, Emerging mutations and associated factors in patients displaying treatment failure on an etravirine-containing regimen. Antivir Ther (2012) 17(1):119–23. doi: 10.3851/IMP1886 22267476

[24] Van Eygen V, Thys K, Van Hove C, Rimsky LT, De Meyer S, Aerssens J, Deep sequencing analysis of hiv-1 reverse transcriptase at baseline and time of failure in patients receiving rilpivirine in the phase iii studies echo and thrive. J Med Virol (2016) 88(5):798–806. doi: 10.1002/jmv.24395 26412111

[25] Vingerhoets J, Azijn H, Fransen E, De Baere I, Smeulders L, Jochmans D, Tmc125 displays a high genetic barrier to the development of resistance: evidence from in vitro selection experiments. J Virol (2005) 79(20):12773–82. doi: 10.1128/JVI.79.20.12773-12782.2005 16188980 PMC1235844

[26] Mackie N Resistance to non-nucleoside reverse transcriptase inhibitors. In: Geretti AM, editor. Antiretroviral resistance in clinical practice. London: Mediscript, Ltd. (2006). 21249773

[27] Lai MT, Lu M, Felock PJ, Hrin RC, Wang YJ, Yan Y, Distinct mutation pathways of non-subtype b hiv-1 during in vitro resistance selection with nonnucleoside reverse transcriptase inhibitors. Antimicrobial Agents chemotherapy (2010) 54(11):4812–24. doi: 10.1128/AAC.00829-10 20805392 PMC2976110

[28] Asahchop EL, Wainberg MA, Sloan RD, Tremblay CL. Antiviral drug resistance and the need for development of new hiv-1 reverse transcriptase inhibitors. Antimicrob Agents Chemother (2012) 56(10):5000–8. doi: 10.1128/AAC.00591-12 22733071 PMC3457356

[29] Rusconi S, Adorni F, Bruzzone B, Di Biagio A, Meini G, Callegaro A, Prevalence of etravirine (Etr)-rams at nnrti failure and predictors of resistance to etr in a large italian resistance database (Arca). Clin Microbiol Infect (2013) 19(10): E443–6. doi: 10.1111/1469-0691.12229 23621421

[30] Cullen BR. Role and mechanism of action of the Apobec3 family of antiretroviral resistance factors. J Virol (2006) 80(3):1067–76. doi: 10.1128/JVI.80.3.1067-1076.2006 16414984 PMC1346961

[31] Goila-Gaur R, Strebel K. Hiv-1 vif, apobec, and intrinsic immunity. Retrovirology (2008) 5:51. doi: 10.1186/1742-4690-5-51 18577210 PMC2443170

[32] Fourati S, Malet I, Lambert S, Soulie C, Wirden M, Flandre P, E138k and M184i mutations in hiv-1 reverse transcriptase coemerge as a result of Apobec3 editing in the absence of drug exposure. AIDS (2012) 26(13):1619–24. doi: 10.1097/QAD.0b013e3283560703 22695298

[33] Jern P, Russell RA, Pathak VK, Coffin JM. Likely role of Apobec3g-mediated G-to-a mutations in hiv-1 evolution and drug resistance. PLoS Pathog (2009) 5(4): e1000367. doi: 10.1371/journal.ppat.1000367 19343218 PMC2659435

[34] McCallum M, Oliveira M, Ibanescu RI, Kramer VG, Moisi D, Asahchop EL, Basis for early and preferential selection of the E138k mutation in hiv-1 reverse transcriptase. Antimicrobial Agents chemotherapy (2013) 57(10):4681–8. doi: 10.1128/AAC.01029-13 23856772 PMC3811420

[35] Mulder LC, Harari A, Simon V. Cytidine deamination induced hiv-1 drug resistance. Proc Natl Acad Sci United States America (2008) 105(14):5501–6. doi: 10.1073/pnas.0710190105 PMC2291111 18391217

[36] Melody K, Roy CN, Kline C, Cottrell ML, Evans D, Shutt K, Long-acting rilpivirine (Rpv) preexposure prophylaxis does not inhibit vaginal transmission of rpv-resistant hiv-1 or select for high-frequency drug resistance in humanized mice. J Virol (2020) 94(8):e01912–19. doi: 10.1128/JVI.01912-19 31969438 PMC7108851

[37] Fletcher P, Harman S, Azijn H, Armanasco N, Manlow P, Perumal D, Inhibition of human immunodeficiency virus type 1 infection by the candidate microbicide dapivirine, a nonnucleoside reverse transcriptase inhibitor. Antimicrobial Agents chemotherapy (2009) 53(2):487–95. doi: 10.1128/AAC.01156-08 19029331 PMC2630639

[38] Asahchop EL, Oliveira M, Wainberg MA, Brenner BG, Moisi D, Toni T, Characterization of the E138k resistance mutation in hiv-1 reverse transcriptase conferring susceptibility to etravirine in b and non-b hiv-1 subtypes. Antimicrob Agents Chemother (2011) 55(2):600–7. doi: 10.1128/AAC.01192-10 21135184 PMC3028807

[39] Schader SM, Oliveira M, Ibanescu RI, Moisi D, Colby-Germinario SP, Wainberg MA. *In vitro* resistance profile of the candidate hiv-1 microbicide drug dapivirine. Antimicrob Agents Chemother (2012) 56(2):751–6. doi: 10.1128/AAC.05821-11 22123692 PMC3264246

[40] Refsland EW, Stenglein MD, Shindo K, Albin JS, Brown WL, Harris RS. Quantitative profiling of the full Apobec3 mrna repertoire in lymphocytes and tissues: implications for hiv-1 restriction. Nucleic Acids Res (2010) 38(13):4274–84. doi: 10.1093/nar/gkq174 20308164 PMC2910054

[41] Picchio GR, Rimsky LT, Van Eygen V, Haddad M, Napolitano LA, Vingerhoets J. Prevalence in the USA of rilpivirine resistance-associated mutations in clinical samples and effects on phenotypic susceptibility to rilpivirine and etravirine. Antiviral Ther (2014) 19(8):819–23. doi: 10.3851/IMP2771 24704709

[42] Das K, Bauman JD, Clark AD Jr., Frenkel YV, Lewi PJ, Shatkin AJ, High-resolution structures of hiv-1 reverse transcriptase/Tmc278 complexes: strategic flexibility explains potency against resistance mutations. Proc Natl Acad Sci United States America (2008) 105(5):1466–71. doi: 10.1073/pnas.0711209105 PMC2234167 18230722

[43] Das K, Clark AD Jr., Lewi PJ, Heeres J, De Jonge MR, Koymans LM, Roles of conformational and positional adaptability in structure-based design of tmc125-R165335 (Etravirine) and related non-nucleoside reverse transcriptase inhibitors that are highly potent and effective against wild-type and drug-resistant hiv-1 variants. J Med Chem (2004) 47(10):2550–60. doi: 10.1021/jm030558s 15115397

[44] Lansdon EB, Brendza KM, Hung M, Wang R, Mukund S, Jin D, Crystal structures of hiv-1 reverse transcriptase with etravirine (Tmc125) and rilpivirine (Tmc278): implications for drug design. J Med Chem (2010) 53(10):4295–9. doi: 10.1021/jm1002233 20438081

[45] Cecilia D, KewalRamani VN, O’Leary J, Volsky B, Nyambi P, Burda S, Neutralization profiles of primary human immunodeficiency virus type 1 isolates in the context of coreceptor usage. J Virol (1998) 72(9):6988–96. doi: 10.1128/JVI.72.9.6988-6996.1998 9696790 PMC109918

[46] Wu L, Martin TD, Vazeux R, Unutmaz D, KewalRamani VN. Functional evaluation of dc-sign monoclonal antibodies reveals dc-sign interactions with icam-3 do not promote human immunodeficiency virus type 1 transmission. J Virol (2002) 76(12):5905–14. doi: 10.1128/jvi.76.12.5905-5914.2002 12021323 PMC136240

[47] Nara PL, Hatch WC, Dunlop NM, Robey WG, Arthur LO, Gonda MA, Simple, rapid, quantitative, syncytium-forming microassay for the detection of human immunodeficiency virus neutralizing antibody. AIDS Res Hum Retroviruses (1987) 3(3):283–302. doi: 10.1089/aid.1987.3.283 3481271

[48] Lee K, Ambrose Z, Martin TD, Oztop I, Mulky A, Julias JG, Flexible use of nuclear import pathways by hiv-1. Cell Host Microbe (2010) 7(3):221–33. doi: 10.1016/j.chom.2010.02.007 20227665 PMC2841689

[49] Melody K, McBeth S, Kline C, Kashuba AD, Mellors JW, Ambrose Z. Low frequency of drug-resistant variants selected by long-acting rilpivirine in macaques infected with simian immunodeficiency virus containing hiv-1 reverse transcriptase. Antimicrobial Agents chemotherapy (2015) 59(12):7762–70. doi: 10.1128/AAC.01937-15 26438501 PMC4649225

[50] Boyer PL, Melody K, Smith SJ, Dunn LL, Kline C, Fischer DK, Two coselected distal mutations in hiv-1 reverse transcriptase (Rt) alter susceptibility to nonnucleoside rt inhibitors and nucleoside analogs. J Virol (2019) 93(11):e00224–19. doi: 10.1128/JVI.00224-19 30894467 PMC6532099

[51] Palmer S, Wiegand AP, Maldarelli F, Bazmi H, Mican JM, Polis M, New real-time reverse transcriptase-initiated pcr assay with single copy sensitivity for human immunodeficiency virus type 1 rna in plasma. J Clin Microbiol (2003) 41(10):4531–6. doi: 10.1128/JCM.41.10.4531-4536.2003 14532178 PMC254331

[52] Palmer S, Kearney M, Maldarelli F, Halvas EK, Bixby CJ, Bazmi H, Multiple, linked human immunodeficiency virus type 1 drug resistance mutations in treatment-experienced patients are missed by standard genotype analysis. J Clin Microbiol (2005) 43(1):406–13. doi: 10.1128/JCM.43.1.406-413.2005 15635002 PMC540111

